# 564. Nirsevimab effectiveness in infants against respiratory syncytial virus (RSV) lower respiratory tract disease (LRTD) and related healthcare utilization

**DOI:** 10.1093/ofid/ofaf695.173

**Published:** 2026-01-11

**Authors:** Amber Hsiao, John R Hansen, Julius Timbol, Lauren D Liao, Bruce Fireman, Ousseny Zerbo, Karine Mari, Christopher Rizzo, William V La Via, Ruvim Izikson, Nicola P Klein

**Affiliations:** Division of Research Kaiser Permanente Vaccine Study Center, Oakland, California; Division of Research Kaiser Permanente Vaccine Study Center, Oakland, California; Kaiser Permanente Vaccine Study Center, Oakland, CA; Kaiser Permanente Vaccine Study Center, Oakland, CA; Division of Research Kaiser Permanente Vaccine Study Center, Oakland, California; Division of Research Kaiser Permanente Vaccine Study Center, Oakland, California; Sanofi Pasteur S.A., Lyon, Auvergne, France; 4Sanofi, Swiftwater, Pennsylvania; Sanofi Pasteur, Swiftwater, Pennsylvania; 4Sanofi, Swiftwater, Pennsylvania; Division of Research Kaiser Permanente Vaccine Study Center, Oakland, California

## Abstract

**Background:**

Respiratory syncytial virus (RSV) is the most common cause of lower respiratory tract disease (LRTD) in infants and young children. In 2023, the monoclonal antibody nirsevimab was FDA approved. ACIP recommends nirsevimab to prevent RSV disease for infants < 8 months during or entering their first RSV season, and for children 8-19 months at high risk of severe RSV entering their second RSV season. We previously reported that nirsevimab was 87.2% effective against RSV LRTD in infants. Here, we describe RSV LRTD incidence since 2016 and report on nirsevimab effectiveness against related healthcare utilization.

**Figure:**
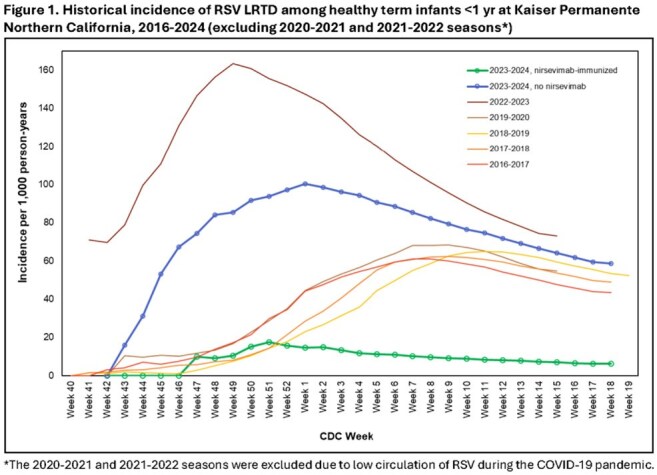

**Figure:**
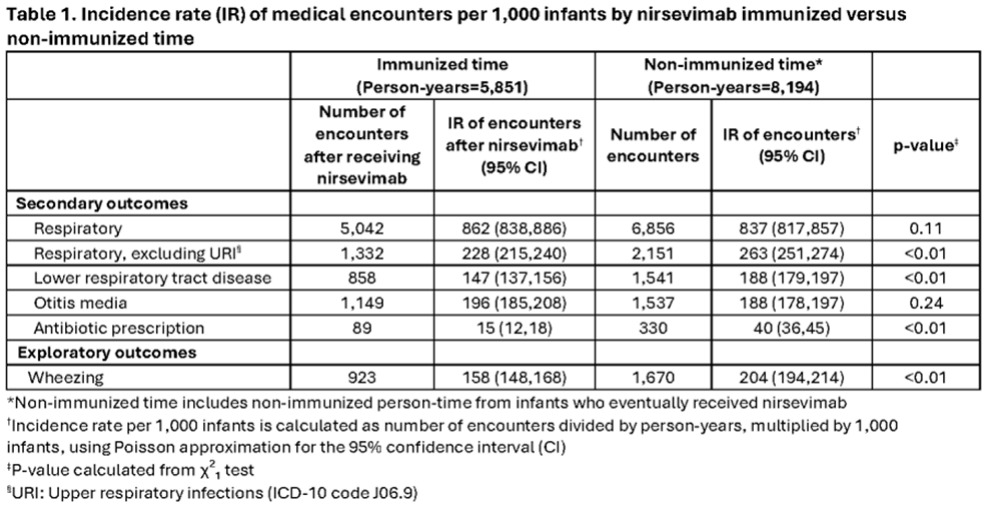

**Methods:**

We included nirsevimab-eligible, healthy term infants born April 2023 to April 2024 at Kaiser Permanente Northern California. RSV LRTD incidence in all healthcare settings among nirsevimab-immunized and non-immunized infants was compared with incidence among healthy term infants born in pre-nirsevimab years (2016-2023). Secondary and exploratory endpoints included other related medical encounters classified by ICD-10 codes or National Drug Codes. For the 2023-2024 cohort, we calculated rates of medical encounters per thousand person-years (PY) of follow-up between nirsevimab administration start (October 19, 2023) or birthdate (if later) through April 30, 2024. Rates of encounters in 6 categories were calculated as total encounters divided by person-years.

**Results:**

The study included 31,900 infants; 15,647 (49%) received nirsevimab. RSV LRTD incidence was 6.1 episodes per 1,000 PY (95% CI: 4.4, 8.5) among nirsevimab-immunized infants. Incidence among non-immunized infants during 2023-2024 was 58.5 episodes per 1,000 PY (95% CI: 53.4, 64.1), similar to pre-nirsevimab years (43.5-73.0) (Figure 1). Rates of antibiotic prescriptions and encounters for respiratory (excluding upper respiratory), LRTD, and wheezing diagnoses were significantly lower among nirsevimab-immunized infants (Table 1).

**Conclusion:**

Nirsevimab greatly reduced the incidence of RSV LRTD during its first season of use and reduced several types of related healthcare utilization.

(Funded by Sanofi and AstraZeneca; ClinicalTrials.gov number, *NCT06325332*)

**Disclosures:**

All Authors: No reported disclosures

